# Genetic Biomarkers as Predictors of Response to Tocilizumab in Rheumatoid Arthritis: A Systematic Review and Meta-Analysis

**DOI:** 10.3390/genes13071284

**Published:** 2022-07-20

**Authors:** Sivakami Janahiraman, Chun Lai Too, Kai Wei Lee, Nor Shuhaila Shahril, Chee Onn Leong

**Affiliations:** 1 School of Postgraduate Studies, International Medical University, Bukit Jalil, Kuala Lumpur 57000, Malaysia; sivakami_5@yahoo.com; 2 Pharmacy Department, Hospital Selayang, Ministry of Health Malaysia, Selayang 68100, Malaysia; 3 Immunogenetic Unit, Institute for Medical Research, National Institutes of Health Complex, Ministry of Health Malaysia, Shah Alam 40170, Malaysia; 4 Department of Pre-Clinical Sciences, Faculty of Medicine and Health Sciences, Universiti Tunku Abdul Rahman, Kajang 31900, Malaysia; lee_kai_wei@yahoo.com; 5 Medical Department, Hospital Putrajaya, Ministry of Health Malaysia, Putrajaya 62250, Malaysia; drshu73@yahoo.com; 6 Centre for Cancer and Stem Cell Research Development and Innovation (IRDI), Institute for Research, International Medical University, Bukit Jalil, Kuala Lumpur 57000, Malaysia; coleong@agtcgenomics.com; 7 AGTC Genomics, Bukit Jalil, Kuala Lumpur 57000, Malaysia

**Keywords:** rheumatoid arthritis, tocilizumab, genetic, polymorphisms, predictor, treatment response

## Abstract

Rheumatoid arthritis (RA) is a lifelong, debilitating disease which incredibly impacts a patient’s quality of life if not treated to the optimal target. The clinical response of tocilizumab, an interleukin-6 (IL-6) inhibitor, is associated with several gene polymorphisms, particularly targeting the IL-6 pathway. This systematic review and meta-analysis seeks to investigate genetic biomarkers that predict the treatment outcome of tocilizumab therapy in RA patients. After evaluating the quality of retrieved records, five studies were chosen to carry out a quantitative synthesis involving 591 participants. We analysed genetic markers of IL-6R single nucleotide polymorphism (SNP)s rs12083537, rs2228145 and rs4329505, FCGR3A, CD69, GALNT18 and FCGR2A. A plausible finding based on meta-analysis revealed that RA patients with homozygous AA genotype for rs12083537 polymorphism of the IL-6R gene demonstrate a better response to TCZ treatment as opposed to homozygous and heterozygous patients with the G allele. Nonetheless, limitations in evaluating the available studies by meta-analysis include a lack of studies with dissimilarities in study design and outcome definitions, small sample sizes with low statistical power and heterogeneity of cohorts, a restricted the number of tested SNPs and small effects for the selected variants. Inconsistent finding remains as a great challenge to forge ahead towards personalised medicine for RA management.

## 1. Introduction

Rheumatoid arthritis (RA) is a multifactorial, autoimmune disease characterised by hyperplastic synovium, cartilage damage, bone erosion, joint destruction with persistent synovitis and systemic inflammation. The onset is triggered by the interaction between environmental factors and genetic predisposition to RA [1,2]. This chronic inflammatory disease affects 0.5–1.1% of the world population, with higher prevalence observed in northern Europe and North America compared to developing countries such as Malaysia [3]. Inflammation in joints leads to swelling, severe pain and stiffness which results in joint damage, and may progress to extra-articular organs, such as the eye, lung and heart. Proinflammatory cytokines, such as tumor necrosis factor (TNF), interleukin (IL)-6, IL-1, IL-8 and granulocyte-macrophage colony-stimulating factor (GM-CSF), and anti-inflammatory cytokines, are both responsible for the chronically activated immune system [4].

Biological disease-modifying antirheumatic drugs (bDMARDs) are highly specific and target a particular pathway of the immune system. Some of these drugs are monoclonal chimeric humanised-fusions antibodies, while others are receptors that have been fused to a part of the human immunoglobulin [5]. Tocilizumab (TCZ) is a novel recombinant humanised-monoclonal antibody which competitively inhibits the binding of IL-6 to its receptors, both in its soluble and membrane-bound forms (sIL-6R and mIL-6R). TCZ has a 15 to 22-fold weaker binding affinity [5]. Chromosome 7 carries the gene encoding IL-6, whereas the gene encoding receptor of IL-6 (IL-6R) can be found on chromosome 1.

TCZ is available as either an intravenous (IV) infusion every 4 weeks or a weekly subcutaneous (SC) injection. It is licensed for moderate-to-severe active RA patients who have had an inadequate response to one or more disease-modifying anti-rheumatic drugs (DMARDs). TCZ can be used either alone or in combination with methotrexate (MTX) or other DMARDs. In patients with an inadequate response to tumor necrosis factor inhibitors (TNFi), switching treatment to TCZ has resulted in significant and sustained improvements in several patient-reported outcomes [6,7,8]. Despite the success of TCZ in the management of patients with RA, data from a pooled analysis reported that 39% of those receiving 8 mg/kg TCZ for 24 weeks lose their initial response, evidenced by failure to achieve American College of Rheumatology (ACR) 20 response [9]. Heterogeneous response to TCZ was observed in RA patients where specific genetic variations were identified as the drug-response biomarkers [10]. Evidence from genome-wide association studies (GWAS) and candidate gene study has identified genetic variants associated with responses to TCZ therapy which may aid to identify patients that are likely to benefit from these biologic therapies [10,11].

Personalised medicine is a rapidly growing field in medicine with a promising solution in the realm of rheumatoid arthritis (RA) for interindividual variability toward drug response. Its aim is to move towards individualised therapy where drugs are selected based on a risk assessment of genomic variants associated with drug response; totally refuting the theoretical assumption of one-size-fits-all in the RA treatment paradigm [12,13]. Pharmacogenomics is the study of variation in human genome that affect the response to drugs. It aims to develop rational means to optimise drug therapy based on the genetic make-up of patients to ensure maximum efficacy with minimal adverse effects, thereby resulting in cost-saving healthcare resources [11]. In RA, a pharmacogenomic approach has been used to identify genetic variants (i.e., single nucleotide polymorphisms, SNP) or genetic signatures that are associated with treatment response. They can be used as genetic predictive biomarkers for patient stratification in relation to treatment response [4].

In this review, we analysed the association between SNPs of five genes and TCZ response in RA patients based on previously-reported candidate gene studies. We conducted a systematic review and meta-analysis to determine the identified genetic factors as potential predictive biomarkers for response towards TCZ amongst RA patients.

## 2. Materials and Methods

### 2.1. Study Design

A systematic review was performed according to the guidelines of the ‘Preferred Reporting Items for Systematic Reviews and Meta-Analysis (PRISMA) statement [14].

### 2.2. Search Strategies

Two individual searches of international literature reviews and studies were carried out using PubMed, Medline Complete, CINAHL Plus, Cochrane Central Register of Controlled Trials and SCOPUS, for clinical trials reporting on treatment response of TCZ from inception until January 2022. A systematic search was done using the MESH terms consisting of several domains: rheumatoid arthritis AND tocilizumab AND genetic AND treatment response. Additionally, we restricted the search to English language papers with full texts. A manual search was done based on cited references from retrieved articles. The details of the search terms have been presented in Supplementary Material Table S1.

### 2.3. Study Selection

Any primary studies that provided genetic variation data in relation to treatment response to TCZ in RA patients were considered for study inclusion. We compared the allelic/genotypic frequencies of the studied genetic variants between TCZ responders and TCZ non-responders. 

### 2.4. Selection Criteria

The inclusion criteria for the studies were as following: (i) Studies will be eligible for inclusion if genetic variants responsible for TCZ treatment response in RA patients have been evaluated; (ii) peer-reviewed studies published in English or have an English translation version; (iii) data will be accepted from cohort, case-control, cross-sectional and intervention studies. On the other hand, studies were excluded if they were: (i) case reports, conference abstracts and reviews; (ii) studies without available allele/genotype frequencies; (iii) contained duplicate data.

### 2.5. Data Extraction 

Two reviewers (SJ and LKW) independently screened for titles and abstracts and then the full text of potentially eligible articles to identify studies fulfilling the inclusion criteria. For each article selected, a reviewer extracted information using a standardised form. The following items were extracted for synthesis: first author’s last name, publication year, study design, number of participants in the study, list of retrieved candidate genes and the treatment outcome measures. The second reviewer confirmed the accuracy of the data extractions. Any disagreement was resolved through adjudication of a third reviewer. 

### 2.6. Study Outcome

The primary outcome was the identification of genetic predictors of TCZ treatment response associated with clinical parameters, such as the Disease Activity Score of 28 Joints (DAS28) CRP/ESR, American College of Rheumatology (ACR)/EULAR 2011 remission criteria, Clinical Disease Activity Index (CDAI) or Simplified Disease Activity Index (SDAI). 

### 2.7. Quality Assessment

Two authors performed the quality assessment independently. The quality of genetic association studies (Q-Genie) tool developed by Sohani et al. was used to assess the quality of the studies included independently by reviewers [15]. Strengthening the Reporting of Genetic Association Studies (STREGA) [16] and Strengthening the Reporting of Genetic Risk Prediction Studies (GRIPS) [17] guidelines were referred to in developing this Q-Genie tool. It comprises nine domains including rationale for study selection and definition of research endpoints, comparability of comparison groups, technical and non-technical classification of the exposure, other sources of bias and control for confounders, sample size and power, a priori planning of analysis, statistical methods, testing of assumptions and inferences for genetic analysis and appropriateness of inferences drawn from results [15] (Supplementary Material Section S1). 

### 2.8. Statistical Analysis

A meta-analysis utilising a random effects model were conducted for all studies with polymorphisms of the same genetic variants (minimum 2 studies) [18]. Heterogeneity of the studies were assessed using I^2^ statistics with a *p*-value < 0.05 considered as significant. An I^2^ values greater than or equal to 75% will be interpreted as evidence of substantial levels of heterogeneity. Odds ratio (OR) and 95% confidence intervals were used as summary statistics to determine the OR of responsiveness to treatment. All analysis were performed with Meta Analyst software using random effect model (the DerSimonian and Laird method) [19]. 

## 3. Results

### 3.1. Systematic Review of Search Results

**Figure 1:**
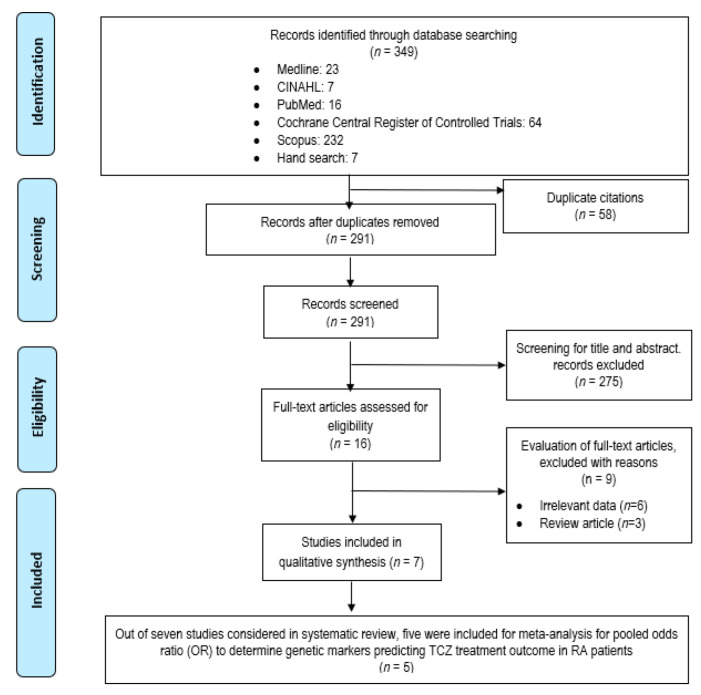
PRISMA flow diagram of study selection process.

Our initial search produced 349 potentially relevant articles and 291 records were retrieved after removing duplicates (Figure 1). A total of 275 articles were excluded due to the discordance with the inclusion/exclusion criteria, resulting in 16 eligible articles for full text screening. After careful evaluation, nine articles were removed for various reasons: irrelevant data (*n* = 6) and review articles (*n* = 3). Eventually, seven articles were included in this current systematic review and only five articles entered the meta-analysis process. 

### 3.2. Study Characteristics

A majority of the studies were cohort studies [20,21,22,23,24] apart from phase 3 clinical trials [9,10] involving five pivotal studies: RADIATE, OPTION, TOWARD, AMBITION and LITHE. These studies were conducted in Spain (*n* = 3) [21,23,24], Denmark (*n* = 1) [20], France (*n* = 1) [22] and another two were multi-centered studies involving several countries. All candidate gene association studies and one genome-wide association study (GWAS) were included in this review. Candidate gene studies are often downplayed by the available or existing knowledge on gene functioning. On the contrary, GWAS studies allow a hypothesis-free search for genetic biomarkers (Supplementary Material Table S2). Six out of seven included papers stated that their patients were receiving the recommended dosage of tocilizumab i.e., 4 or 8 mg/kg every 4 weeks intravenously or 162 mg every 4 weeks subcutaneously until the response evaluation time points.

Wang et al. [10] was the first to pave the way in exploring predictive genetic biomarkers specific for TCZ response by adopting a GWAS approach in a population study with a cohort of 1683 back in 2013. This large-scale study involving various randomised clinical trials and international research collaborations yielded eight novel pharmacogenetic loci for TCZ treatment response in RA, which includes SNPs in the coding region of GALNT18 (rs4910008), ENOX1 (rs9594987), CLEC2D (rs1560011), CD69 (rs11052877), KCNMB1 (rs703505) and SLC9A7 (rs7055107) genes, as well as the rs10108210 and rs703297 (non-gene) variants. As to validate this finding, confirmation with an independent cohort study was conducted by Mar Maldona-do-Montoro et al. [24] with a cohort of 79 Caucasians. Having in mind the main study limitation of a small sample size, a conclusion was drawn about the GALNT18 C-allele and the CD69 A-allele as potential predictors of a good response to TCZ in RA. This association was replicated by Luxembourger, C. et al. [22] who carried out a retrospective, prospective cohort study using two different French cohorts of 154 and 60 patients to investigate whether SNPs in 21 candidate genes (Table 1) were associated with TCZ responsiveness. Surprisingly, only one strong association was established between IL6R polymorphism (rs12083537) and the TCZ treatment response in both cohorts.

Four studies had small sample sizes (less than 100 participants) whereas the other three studies consisted of various sample sizes (150–1700 participants). The subjects in Wang et al.’s [9] candidate gene study is part of the same cohort with the GWAS study published by the same author in the same year [10]. Likewise, subjects in Mar Maldonado-Montoro et al.’s study [23] are a subset of the study published earlier in 2016 [24]. All studies reported the ethnicity of their participants; a large number of them were Caucasians. In addition, studies by Wang et al. included patients of various ethnicities, from East Asian, Southeast Asian, South African, Western European, North and South American to the Latin American population [10]. Blood [10,20,22,25] and saliva [21,23,25] were the two most common biospecimen used for genotyping analysis. 

Most of the studies employed multiple genotyping assays. The most common assay techniques used were a real-time PCR TaqMan genotyping assay, allele-specific kinetic PCR analysis, IIlumina Bead-Chip arrays and a bead-based assay (Luminex platform). All the studies assessed compliance with the Hardy–Weinberg equilibrium, and sample quality control was conducted prior to genotyping. Among these seven studies, only five studies provided full distribution of SNP genotypes. 

Studies were stratified by measure of clinical response or variables; with all seven studies adopting the 28-joint Disease Activity Score (DAS28), which comprised tender joint count (TJC) and swollen joint count (SJC) of 28 specified joints, inflammatory markers of erythrocyte sedimentation rate, (ESR)/C reactive protein (CRP) and patient global assessment using a 100 mm visual analogue scale (VAS) as their primary endpoint. On the other hand, a study by Enevold, C. et al. [20] reported DAS28 as a secondary endpoint instead because SJC was regarded as the primary clinical outcome parameter owing to its reliability and relevancy in clinical effect. 

The effectiveness of treatment was measured by tracking the change in DAS28 (∆DAS28) from baseline until time elapsed before the evaluation of response. It is noteworthy that three studies categorised treatment outcome as satisfactory (present DAS28 < 3.2 and DAS28 improvement > 1.2) and unsatisfactory (present DAS28 ≥ 3.2 and DAS28 improvement ≤ 1.2); meanwhile, the other four studies dichotomised into responders (good and moderate response of ≤1.2–>1.2) and non-responders (no response of ≤ 0.6) based on the EULAR response criteria. There were also inconsistencies in clinical end points evaluation where some have assessed the TCZ response outcome as early as 3 months [20,22] whereas the rest of them have made the assessment at a different timepoints, i.e., at 4, 6, 12 and 18 months. 

An overview of the main characteristics of the studies included are summarised in Table 1. Patient characteristics are presented in Supplementary Material Table S3. 

### 3.3. Quality Assessment

Among all the reviewed studies, four were scored to have high quality (mean score of >40 for studies without control group), two were categorised as moderate quality and one study was rated as poor quality (Supplementary Material Section S2). Quality analysis for the seven studies is shown in Supplementary Material Section S2. On average, most of the studies assessed were judged to be of good quality, and fulfilled the requirement of mean scores of >3 for most of the items on the Q-Genie tool except for the domain ‘sample size and power’. All studies did not report the sample size calculation. As a consequence, studies’ populations were often too small, more so when the sub-analyses were carried out per SNP. However, all the papers reported sufficient general and basic characteristics to get a sense of the population study. TCZ dose was described in all studies except for one study by Luxembourger et al. [22]. Previous exposure to biological therapy was illustrated in three studies only; two studies by Maldonado-Montoro et al. and one study by Morales et al. [21,23,24]. The use of biologic monotherapy or combination therapy was explicitly stated in all studies and unreported in one study authored by Enevold, C. et al. [20]. Two studies excluded patients who could not complete the required follow-up period due to treatment abandonment because of lack of effectiveness, leading to possible bias with non-responders being more likely to drop out early. Many of the studies did not report how missing data were handled (42.8%); and complete descriptions of planned analysis was not sufficiently described in more than half (57.1%) of the papers. The type of analysis used in the main comparison was unreported in one study [10], a per-protocol analysis was used in four studies [21,23,24,25] and an as-treated/complete case analysis in two studies [20,22].

### 3.4. Genetic Markers Associated with TCZ Treatment Response

In total, 26 polymorphisms were investigated, including polymorphisms in the following 23 candidate genes: interleukin 6 receptor (IL-6R); Fc fragment of IgG receptor 3A (FCGR3A); cluster of differentiation 69 (CD69); polypeptide N-acetylgalactosaminyltransferase 18 (GALNT18); C-type lectin domain family 2 member D (CLEC2D); ecto-NOX disulphide-thiol exchanger 1 (ENOX1); kv channel-interacting protein 1 (KCNIP1); polypeptide N-acetylgalactosaminyltransferase 4 (GALNTL4); solute carrier family member A7 (SLC9A7); Fc fragment of IgG receptor 2A (FCGR2A); cluster of differentiation 84 (CD84); Fc fragment of IgG receptor 3B (FCGR3B); Fc fragment of IgG receptor 2B (FCGR2B); protein tyrosine phosphatase, receptor type C (PTPRC); interleukin 10 (IL10); tumor necrosis factor (TNF); IL6; TNF receptor superfamily member 10A (TNFRSF10A); tumor necrosis factor receptor–associated factor;TRAF1 and complement component 5,C5 (TRAF1/C5); TNF receptor superfamily member 1A (TNFRSF1A); protein tyrosine phosphatase non-receptor Type 2 (PTPN2); Lymphotoxin α (LTA) and transforming growth factor β 1 (TGFB1). The polymorphisms identified in candidate gene studies in relation to the outcome from TCZ treatment of patients with RA are shown in Supplementary Material Table S3. Seven polymorphisms (studied in at least two studies) with data of genotypes and treatment response retrieved completely were selected for meta-analysis. Table 2 depicts the meta-analysis results for seven polymorphisms in five genes (IL-6R rs12083537A/G, IL-6R rs2228145A/C, IL-6R rs4329505A/G, FCGR3A rs396991G/T, CD69 rs11052877A/G, GALNT18 rs4910008C/T and FCGR2A rs1801274C/T).

### 3.5. TCZ-Response Related Polymorphism

**Figure 2:**
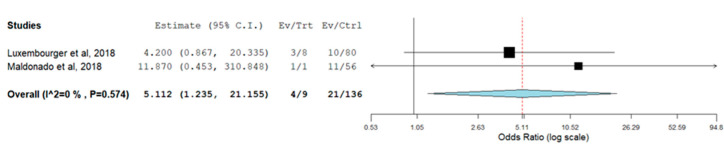
Forest plot of IL-6R rs 12083537 (genotype AA as control vs. genotype GG as testing/risk genotype) and response to TCZ treatment [22,23] (Ev/Trt refers to number of participants not responding to the treatment in the cohort of GG genotype carrier. Ev/Ctrl refers to number of participants not responding to the treatment in the cohort of AA genotype carrier).

**Figure 3:**
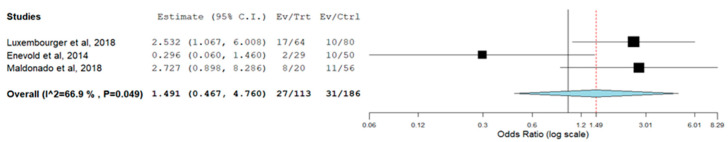
Forest plot of IL-6R rs 12083537 (genotype AA as control vs. genotype AG as testing/risk genotype) and response to TCZ treatment [20,22,23] (Ev/Trt refers to number of participants not responding to the treatment in the cohort of AG genotype carrier. Ev/Ctrl refers to number of participants not responding to the treatment in the cohort of AA genotype carrier).

**Figure 4:**
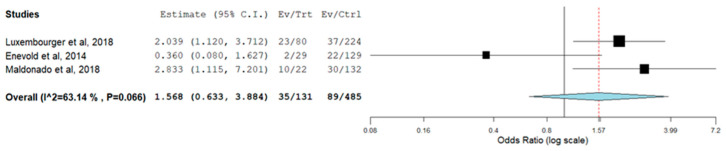
Forest plot of IL-6R rs 12083537 (allele A as control vs. allele G as risk allele) and response to TCZ treatment [20,22,23] (Ev/Trt refers to number of participants not responding to the treatment in the cohort of G allele carrier. Ev/Ctrl refers to number of participants not responding to the treatment in the cohort of A allele carrier).

The meta-analysis of IL-6R rs12083537 genotypic model showed that individuals who carry IL-6R rs12083537 GG genotype are significantly associated with a non-response to TCZ treatment with a pooled odds ratio of 5.112 (95% CI = 1.235, 21.155) compared to those carry IL-6R rs12083537 AA genotype (Figure 2). On the other hand, IL-6R rs12083537 AG genotype carriers were found to be not significantly associated with a poor response to treatment (pooled OR = 1.491, 95% CI = 0.467, 4.760) (Figure 3). Further analysis using the allelic model demonstrated that the IL-6R rs12083537 minor allele G was non-significantly associated with non-response to TCZ treatment, as compared to the IL-6R rs12083537 major allele A (pooled OR = 1.568, 95% CI = 0.633, 3.884) (Figure 4). 

The presence of allele A is a predictor of good response to TCZ treatment. Our analysis showed that RA patients with one or two copies of the G allele were not responding to the TCZ treatment. However, this finding should be interpreted with caution as only two studies were included for meta-analysis. 

Apart from IL-6R rs12083537, we also performed meta-analysis for the SNPs i.e., IL-6R rs2228145, IL-6R rs4329505, FCGR3A rs396991, CD69 rs11052877, FCGR2A rs1801274 and GALNT-18 rs4910008. None of the analysed SNPs were found to be associated with TCZ treatment response (Supplementary Material Section S3, A to R). It is noteworthy that this finding should be considered as in a premature stage to permit meaningful comparison. 

## 4. Discussion

### 4.1. Main Findings

To the best of our knowledge, this is the first and only systematic review and meta-analysis investigating the predictive genetic biomarkers and clinical response to TCZ, an IL-6R blocker used for treating RA.

We encountered a total of seven studies investigating twenty-three gene candidates. In meta-analysis, we examined associations between these seven gene polymorphisms and TCZ response, which provides evidence of a significantly increased TCZ treatment response of IL-6R rs12083537 AA genotype in RA patients. 

Results from Maldonado-Montoro et al. [23] and Luxembourger, C. et al. [22] reinforce the potential genetic ability of A-allele of rs12083537 with a better response to TCZ. Maldonado-Montoro et al. [23] demonstrated that RA patients harbouring the AA-genotype for rs12083537 fare better in terms of LDA after 12 months of TCZ therapy (OR: 13.0; CI 95%: 2.31, 72.91; *p* = 0.004). Similarly, Luxembourger et al. [22] reported that patients with the homozygous AA-genotype exhibit a significantly better EULAR response after 3 months of treatment. This finding from the first cohort of retrospective study was replicated in the second prospective trial which yielded the same result [22].

It is noteworthy that there are inconsistencies in clinical end points in the aforementioned studies whereby the TCZ-response outcome was measured at 12 months (low disease activity/remission) in Maldonado-Montoro et al.’s [23] study as opposed to a very early assessment within 3 months (primary response) by Luxembourger, C. et al. [22]. The duration of TCZ exposure and dosage taken would have a bearing on the clinical end points apart from the inclusion of prospective studies with a longer follow-up period which may enhance the accuracy of the findings. In addition, EULAR-response status also varies between these two studies. 

On another note, a few other studies generate conflicting results. A distinctively large phase-3-controlled clinical trial of more than 3700 patients by Wang et al. [9] discovered no relationship between genetic polymorphisms in IL6 or IL-6R with treatment response to TCZ. However, it is noted that SNP rs12083537 was not investigated in this study. Conversely, Enevold et al. [20] reported that the major allele (A) of rs12083537 and the minor allele (C) of rs4329505 were associated with poor response towards swollen joint count. Furthermore, the AAC haplotype for rs12083537, rs2228145 and rs4329505 of IL6R was strongly associated with a poorer response to TCZ based on the swollen joint count (*p* = 0.00004) and with borderline significance of the EULAR response (*p* = 0.05). More studies are warranted to obtain a robust relationship between IL-6R rs12083537 and TCZ outcomes in RA patients. 

### 4.2. IL6R Gene Polymorphism as Predictor Response to TCZ

This study reveals an association between SNP rs12083537 (A > G) and response to TCZ in patients with RA. IL-6R rs12083537 can be found on chromosome 1 within intron 1, 2.9 kb away from exon 1, which varies by the alternative presence of an adenine or a guanine. 

Based on a previous study of asthmatic patients, the IL6R gene transcription was not altered by the SNP rs12083537 gene polymorphism as there was no significant relationship found between rs12083537 and IL6R mRNA levels [25]. However, the same authors mentioned the possibility of rs12083537 being a regulatory variant for soluble IL-6R (sIL-6R) serum levels exerting a functional effect. In fact, they showed a relationship between rs12083537 and sIL-6R levels indicative of an epigenetic regulation of expression. In RA, a mechanistic study reported that IL-6R inhibition by TCZ resulted in an increased level of serum IL-6 and serum sIL-6R until a steady state [26]. It is evident that the formation of TCZ/sIL-6R immune complex reduces the half-life for sIL-6R elimination. This fact is supported by a recent study which reported a correlation between clinical response to TCZ and baseline IL-6/sIL-6R levels in RA patients [27]. Suffice to say that this observation supports the relationship between SNP IL-6R rs12083537 and TCZ response in RA.

IL-6 is a pleiotropic proinflammatory cytokine produced by various types of cells as a result of on-going infection, trauma and immunologic challenges including autoimmune diseases [28]. High concentrations of IL-6 are predominantly found, not only in the synovial fluid, but also in the sera of patients with RA which are responsible for the systemic features of RA. IL-6 involvement in RA pathogenesis includes B-cell proliferation, matrix metalloproteinase expression, acute-phase response and anaemia [29]. Severe RA correlates with thrombocytosis, hypergammaglobulinemia and elevated ESR and CRP levels in parallel with plasma and synovial levels of IL-6 [30]. In fact, high levels of CRP are one of the predictors for poor outcome among RA patients. Presence of IL-6 in bone marrow leads to systemic and periarticular bone loss [30].

There are two pathways in IL-6R signal transduction namely the classical (cis-) or a trans-signalling pathway. IL-6 binds to mIL-6R and forms a trimer with glycoprotein 130 (gp130) in the cis-signalling pathway. The dimerisation of this heterotrimer with another IL-6/mIL-6R/gp130 complex forms a signalling complex [31]. Nonetheless, mIL-6R expression is largely confined to a subgroup of leucocytes and hepatocytes. Hence, the trans-signalling pathway enables the sIL-6R to bind with IL-6, forming a complex that triggers dimerisation of membrane-bound gp130 and induces responses on cells that do not express the mIL-6R [28]. Soluble glycoprotein 130 (Sgp130) is the ubiquitously expressed antagonist of the IL-6/sIL-6R complex that selectively inhibits IL-6 signalling which is produced when the gene gp130 is spliced [32].

Apparently, serum plasma levels of IL-6 and IL-6R may vary between RA patients [28]. Some of this heterogeneity is attributed to genetic make-up. The IL-6R rs2228145 (previously known as IL-6R rs8192284) (A > C) polymorphism is present at the cleavage site of mIL-6R (Gln 357/Asp358) and has been associated with increased sIL-6R levels and RA susceptibility [33]. This SNP is found in exon 9 of IL6R on chromosome 1 and is carried by approximately 40% of the Scandinavian population. It has been related with a variety of diseases, including type 2 diabetes [34]. Two other SNPs, rs12083537 (A > G) and rs4329505 (T > C), located in intron 1 and intron 9 of IL6R, respectively, are closely linked with altered levels of circulating C-reactive protein (CRP) [35]. Meanwhile, an IL-6 rs1800795-174 G/C promoter gene polymorphism influences the transcriptional activity leading to changes in serum levels of IL-6 [36].

### 4.3. Study Strengths and Limitations

This review has its own limitations owing to the diversity of studies available on TCZ pharmacogenetics which posed a great challenge in performing pooled or meta-analysis. There were a lack of pharmacogenetic studies on TCZ responses with dissimilarities in study design, significant phenotype heterogeneity (i.e., presence of poor prognostic factors, such as rheumatoid factor/anti-citrullinated peptide autoantibodies, high disease activity, early erosion, failure of two or more csDMARDs), sample sizes, different timeline of outcome measurements, various TCZ dosing regime and duration of exposure, background therapy and ethnic variability to permit comparison between studies and thus considerably affect the treatment outcomes. Moreover, none of the eligible studies performed subsets analysis based on the prognostic factors; therefore, the assessment of patients with poor response to therapy due to presence of prognostic factors was not possible for this review. 

Initial findings of 26 SNPs from seven studies limited the ability to perform meta-analysis as most of the SNPs were only investigated once. Moreover, inconsistence and inadequate result reporting between studies restricted meta-analysis to two to three studies in this study. Even though most of the studies were designed retrospectively, it certainly lacked a power calculation; thus, it is unable to rule out the possibility of distorted results of meta-analysis due to underpowered studies. Understanding the nature of biomarkers with a strong effect to be utilised in clinical practice can be sometimes jeopardised by weak and small effect size of the pharmacogenetic studies. It can also be further complicated by the presence of other biomarkers, such as clinical biomarkers (e.g., high baseline ESR, high baseline CRP and high baseline DAS28-ESR scores), transcriptomic biomarkers (e.g., expression of Type 1 interferon (IFN) response gene (IFI6, MX2 and OASL) and Metalllothionein 1G (MT1G) genes) and serum biomarkers (e.g., serum D-dimer, IL-1β levels, serum 14-3-3η levels and serum gp130 levels), which are reported to be associated with response to tocilizumab [32]. Nevertheless, it is noteworthy that small studies are crucial to explore new avenues in a relatively new field of research as it holds promise for the value of pharmacogenetics in RA treatment.

EULAR criteria were employed in all studies to assess the clinical or treatment response variables. However, there was no uniformity in terms of the definition for responders and non-responders in between the studies. TCZ responsive outcomes measured at various time points i.e., 3, 4, 6, 12 and 18 months between studies made the comparison between treatment outcomes more complicated. The use of TCZ in combination with other DMARDs can also influence the outcome measures. The dose of TCZ prescribed varied, and some did not use the maximum dose compared to other studies which did, which may have a direct effect on TCZ treatment response.

Finally, most of the studies on genetic predictors and drug response have included data from mainly the Caucasian populations. The relative significance of polymorphism of the IL-6 receptor in drug response may be based on ethnicity as the allele frequency of the polymorphism may differ between various ethnic populations. Hence, generalising the pharmacogenetic findings of TCZ responsiveness to the RA population from different ethnic populations across the world warrants future research towards precise medicine and RA care management. 

## 5. Conclusions

This review suggests that there is a plausible association between IL-6R rs12083537 (A > G) polymorphism in RA. However, this inference warrants careful consideration in determining the SNP specificity with TCZ treatment as the existing evidence is limited and too heterogenous for a significant quantitative analysis. Replications of this finding is required to improve the strength of the current review. Multicentred, multi-ethnic with larger sample size, prospectively designed with clinically relevant and unified outcome measures of TCZ response, apart from dosing regime and background therapy with a longer follow-up duration, will be able to give a better insight into the discovery of a promising predictive genetic biomarker in determining TCZ therapeutic response. 

## Figures and Tables

**Table 1:** Study characteristics of included research articles for review.

Study [Refs]	Year	Study Approach	Study Design	Sample Size	Genes (& SNP) Investigated	Outcome Measures	
						Response Criteria	Response Evaluation
Wang J et al. [10]	2013	GWAS	Multicentre trial (6 randomized, controlled clinical studies)	1683	CD69.rs11052877, GALNTL4.rs4910008, ENOX1.rs9594987, CLEC2D.rs1560011, KCNIP1.rs703505, rs10108210, rs703297 SLC9A7.rs7055107	∆DAS28	4 and 6 months
Wang J et al. [9]	2013	Candidate genes	Multicentre trial (5 randomized, controlled clinical studies)	927	26 gene polymorphisms of IL6R and IL-6	∆DAS28	Baseline and 4 months
Enevold et al. [20] *	2014	Candidate genes	Retrospective cohort	79	rs12083537, rs2228145, rs4329505 gene polymorphisms of IL6R	EULAR/ ∆DAS28	3 months
Mar Maldonado-Montoro et al. [24] *	2016	Candidate genes	Retrospective cohort	79	CD69.rs11052877, GALNT18.rs4910008, CLEC2D.rs1560011, KCNMB1.rs703505, ENOX1.rs9594987, rs10108210 & rs703297	EULAR/ ∆DAS28	6 and 18 months
Jimenez Morales et al. [21] *	2019	Candidate genes	Retrospective prospective cohort	87	FCGR2A.rs1801274, FCGR3A.rs396991	EULAR/ ∆DAS28	6,12 and 18 months
Mar Maldonado-Montoro et al. [23] *	2018	Candidate genes	Retrospective cohort	77	rs12083537, rs2228145, rs4329505, rs11265618 gene polymorphisms of IL6R	EULAR/ ∆DAS28	12 months
Luxembourger et al. [22] *	2019	Candidate genes	Retrospective cohort & prospective, open, multicentre trial	154 & 60	IL6R, CD84, FCGR2A, FCGR3A, FCGR3B, FCGR2B, PTPRC, IL10, KCNIP1, TNF, IL6, TNFRSF10A, TRAF1/C5, GALNT18, TNFRSF1A, CD69, PTPN2, LTA, TGFB1, and SLC9A7	EULAR/ ∆DAS28	3 months

* Refs [20,21,22,23,24] were included into meta-analysis. SNPs that have been analysed in meta-analysis are IL-6R.rs12083537, IL-6R.rs2228145, IL-6R.rs4329505, FCGR3A.rs396991, CD69.rs11052877 and GALNT18.rs4910008.

**Table 2:** Summary of meta-analysis results.

Candidate Gene	SNPS	Genotype/Allele	Categories	OR	95% CI	I^2^	*p*-Values
Il-6R	rs12083537	*Genotype*	GG	5.112	1.235, 21.155	0.0	0.574
			AG	1.491	0.467, 4.760	66.9	0.049
			AA	Reference	-		
		*Allele*	G	1.568	0.633, 3.884	63.14	0.066
			A	Reference			
Il-6R	rs2228145	*Genotype*	CC	0.789	0.220, 2.835	0.0	0.469
			CA	0.945	0.405, 2.205	0.0	0.376
			AA	Reference	-		
		*Allele*	C	0.336	0.088, 1.292	66.35	0.085
			A	Reference	-		
Il-6R	rs4329505	*Genotype*	GG	2.505	0.423, 14.842	0.0	0.584
			GA	1.685	0.728, 3.905	0.0	0.336
			AA	Reference	-		
		*Allele*	G	1.665	0.827, 3.350	10.55	0.290
			A	Reference	-		
FCGR3A	rs396991	*Genotype*	GG	0.509	−0.487, 1.505	0.0	0.963
			GT	1.898	0.547, 6.586	58.52	0.120
			TT	Reference	-		
		*Allele*	G	1.319	0.843, 2.065	0.0	0.812
			T	Reference	-		
CD69	rs11052877	*Genotype*	GG	3.470	0.642, 18.765	67.73	0.078
			GA	1.570	0.505, 4.883	50.22	0.156
			AA	Reference	-		
		*Allele*	G	1.807	0.788, 4.142	70.35	0.066
			A	Reference	-		
GALNT18	rs4910008	*Genotype*	TT	1.284	0.499, 3.305	4.96	0.305
			TC	1.579	0.231, 10.800	76.92	0.037
			CC	Reference	-		
		*Allele*	T	1.070	0.690, 1.660	0.0	0.448
			C	Reference	-		
FCGR2A	rs1801274	*Genotype*	CC	0.618	0.060, 6.397	56.7	0.129
			CT	0.890	0.347, 2.283	0.0	0.571
			TT	Reference	-		
		*Allele*	C	0.722	0.205, 2.550	72.36	0.057
			T	Reference	-		

(Ev/Trt refers to number of participants not responding to the treatment in the cohort of GG genotype carrier. Ev/Ctrl refers to number of participants not responding to the treatment in the cohort of AA genotype carrier). (Ev/Trt refers to number of participants not responding to the treatment in the cohort of AG genotype carrier. Ev/Ctrl refers to number of participants not responding to the treatment in the cohort of AA genotype carrier). (Ev/Trt refers to number of participants not responding to the treatment in the cohort of G allele carrier. Ev/Ctrl refers to number of participants not responding to the treatment in the cohort of A allele carrier).

## Data Availability

All data used in this review are included in the main text and Supplementary Material.

## Supplementary Materials

The following are available online at https://www.mdpi.com/article/10.3390/genes13071284/s1, Supplementary Material Table S1: Search strategies, Supplementary Material Section S1: Quality of genetic association studies (Q-Genie), Supplementary Material Table S2: Patient characteristics of the included studies, Supplementary Material Section S2: Quality assessment for the included original studies, Supplementary Material Table S3: Polymorphisms that were significantly associated with Tocilizumab response in at least one candidate gene study. The results from all available candidate gene studies are presented per variant, including conflicting results and Supplementary Material Section S3 (A to R): Meta-analysis results.
