# Supplementary material for: Genetic Biomarkers as Predictors of Response to Tocilizumab in Rheumatoid Arthritis: A Systematic Review and Meta-Analysis
Source: Genes (Basel). 2022 Jul 20;13(7):1284. doi: 10.3390/genes13071284 (PMC9323282; doi:10.3390/genes13071284)
Supplement: Supplementary file 1 [file genes-13-01284-s001.zip › genes-1748959-supplementary.pdf]

Supplementary Information

Supplementary Material Table S1: Search strategies

Alternative names for medicine		Alternative names for genetic variation		Alternative names for illness		Alternative names for outcome
Interleukin-6 blockers OR IL-6 blockers OR interleukin-6 inhibitor OR IL-6 anti-Interleukin-6 OR anti IL-6 OR interleukin-6 receptor inhibitors OR IL-6 receptor inhibitors OR Tocilizumab OR TCZ OR Actemra	AND	Polymorphisms OR polymorphism OR genetic OR genetic variants OR genetic variant OR pharmacogenomics OR pharmacogenomic OR pharmacogenetics OR pharmacogenetic OR DNA polymorphisms OR DNA polymorphism OR deoxyribonucleic acid polymorphism OR deoxyribonucleic acid polymorphisms OR gene polymorphism OR gene polymorphisms OR genetic polymorphism OR genetic polymorphisms OR Single nucleotide polymorphism OR Single nucleotide polymorphisms OR Candidate gene association OR Candidate genes association OR Candidate gene associations OR Candidate genes associations OR Polymorphism single nucleotide OR Genetic variation OR Genetic variation OR genome-wide association study OR genome-wide association OR Genome-Wide Association Studies OR Genome Wide Association Scan OR Genome Wide Association Studies OR GWA Study OR GWA Studies OR GWAS OR Whole Genome Association Analysis OR Whole Genome Association Study OR Genome Wide Association Analysis OR Genome Wide Association Study OR Genome Wide Association Studies OR Genetic association study OR Genetic association studies OR Genetic association	AND	rheumatoid arthritis OR arthritis rheumatoid	AND	therapeutic response OR therapeutic responses OR treatment response OR treatment responses OR drug response OR drug responses OR clinical response OR clinical responses OR response OR responses OR efficacy OR efficacies

Supplementary Material Section S1: Quality of genetic association studies (Q-Genie)

Please indicate your selection for each question as follows:

☐ 1	☐ 2	X 3	☐ 4	☐ 5	☐ 6	☐ 7
Poor		Good		Very Good		Excellent

1. Rationale for study

Please rate the study on the adequacy of the presented hypothesis and rationale.

When rating the study, please consider the following:

- *Was a scientific rationale for chosen genes presented to avoid selective reporting of positive results?*
  If this is a GWAS design, where a hypothesis-free approach is taken, a rationale for selecting this design should be presented.

☐ 1	☐ 2	☐ 3	☐ 4	☐ 5	☐ 6	☐ 7
Poor		Good		Very Good		Excellent

2. Selection and definition of outcome of interest. The outcome can be cases/disease status or a quantitative trait.

Please rate the study on the classification of the outcome (e.g. disease status or quantitative trait).

When rating the study, please consider the following:

- *Were the cases appropriately defined?*
  Outcome definitions will vary from independent adjudication or reliable laboratory measures (strong) to self-report (moderate) to no-description (poor)
- *Were participants appropriately sampled?*
  Participants should be sampled in a way to avoid selection bias as appropriate to the study objectives (e.g. such as selecting the most sick cases if the objective is not to enrich cases). Included participants should reflect the entire population of interest.
- *Were the case/outcome assessors blinded to the genotype status?*
- *If applicable, was follow-up length appropriate for outcome to occur and was the attrition rate acceptable?*

☐ 1	☐ 2	☐ 3	☐ 4	☐ 5	☐ 6	☐ 7
Poor		Good		Very Good		Excellent

3. Selection and comparability of comparison groups (if applicable)

Please rate the study on appropriateness of comparison groups (e.g. control groups).

When rating the study, please consider the following:

- *Were the controls appropriately defined?*
- *Were the controls sampled in a way to minimize selection bias?*
- *Was a detailed description of selection procedure (i.e. eligibility criteria, sources and methods of ascertainment, methods of matching if applicable) outlined or referenced?*
- *Were the assessors of control status blinded to the genotype status?*

Please note: In multi-ethnic studies, allele frequencies and disease risks may differ. Consequently, confounding may occur if these sub-populations are unevenly distributed across exposure groups (or between cases and controls); therefore, details of the sub-populations (e.g. ethnicity) should be reported.

☐ 1 ☐ 2 ☐ 3 ☐ 4 ☐ 5 ☐ 6 ☐ 7

Poor Good Very Good Excellent

4. Technical classification of the exposure

Please rate the study on the technical classification of the genetic variant.

When rating the study, please consider the following:

- *Was the source (e.g. buffy coat) and method of storage for the DNA sample appropriate?*
- *Were the methods of DNA ascertainment similar for comparison groups (if applicable)?*
- *Was the genotyping platform and allele-calling algorithm appropriate?*
- *Were the genotyping error & call rates appropriate? Call rates below 95% indicate poor genotyping quality.*
- *Were the genotype call rates and SNP missingness similar between the comparison groups?*
- *Was agreement with the Hardy Weinberg equilibrium tested in controls?*
- *If applicable, did the authors check for samples with outlying heterozygocity to assess quality of genotyping?*

Please note: if genotypes are imputed, authors should describe methods and rationale for imputing

☐ 1 ☐ 2 ☐ 3 ☐ 4 ☐ 5 ☐ 6 ☐ 7

Poor Good Very Good Excellent

5. Non-technical classification of the exposure

Please rate the study on the non-technical classification of the genetic variant.

When rating the study, please consider the following:

- *Did a blinded assessor conduct the genotyping?*
- *Was genotyping conducted in all the participants from the study simultaneously or in smaller batches? If so, were methods across batches same?*

- *If applicable, were samples randomized prior to genotyping (e.g. not all controls on one plate and cases on another)?*

☐ 1	☐ 2	☐ 3	☐ 4	☐ 5	☐ 6	☐ 7
Poor		Good		Very Good		Excellent

6. Other sources of bias

Please rate the study on the disclosure and discussion of sources of bias.

In addition to selection and classification bias previously discussed, many other potential sources of bias exist (e.g. time-lag bias, attrition bias, et cetera). Please consider whether all sources of bias were disclosed and their effect on the results discussed.

☐ 1	☐ 2	☐ 3	☐ 4	☐ 5	☐ 6	☐ 7
Poor		Good		Very Good		Excellent

7. Sample size and power

Please rate whether the study was adequately powered.

- *Was the sample size appropriate?*
- *Was an a priori power analysis conducted?*

☐ 1	☐ 2	☐ 3	☐ 4	☐ 5	☐ 6	☐ 7
Poor		Good		Very Good		Excellent

8. A priori planning of analyses

Please rate the study on the planned analyses.

- *Was the analysis plan appropriate and sufficiently described?*
- *Was selective and/or inappropriate reporting avoided (i.e. all results from tests conducted were reported)? Authors should identify where additional results can be found if not included in the primary paper (e.g. supplementary tables).*
- *Were the tested subgroups, interactions, and sensitivity analyses described and reported?*
- *Was the statistical software used identified?*

☐ 1	☐ 2	☐ 3	☐ 4	☐ 5	☐ 6	☐ 7
Poor		Good		Very Good		Excellent

9. Statistical methods and control for confounding

Please rate the study on statistical methods.

- *Were important confounders appropriately controlled?*

- *Were missing data for samples and genetic variant was appropriately handled? >10% missing genotype data is often unacceptable.*
- *Were the results adjusted for multiple testing to avoid false positive results? Please note this is particularly important in analyses of large datasets.*

Please note: For multiethnic studies or those with sub-populations, statistical methods, such as principle components analysis, should control for presence of resultant confounding.

1	2	3	4	5	6	7
Poor		Good		Very Good		Excellent

10. Testing of assumptions and inferences for genetic analyses

Please rate the study on the description and test of all assumptions and inferences.

- *Were all assumptions concerning the genetic analysis tested? Specifically,*
  - *i) Haplotypes may be inferred as a result of lack of availability of family data. Numerous methods exist for inferring haplotypes; authors should specifically report how this inference was made.*
  - *ii) In non-family based studies, some individuals may be distantly related or part of a consanguineous group, which may lead to inaccurate results and should be tested with appropriate measures.*
  - *iii) Reported sex and ethnicity should also be checked prior to conducting analyses.*

1	2	3	4	5	6	7
Poor		Good		Very Good		Excellent

11. Appropriateness of inferences drawn from results

Please rate the study on whether conclusions drawn by the authors were supported by the results and appropriate methods.

1	2	3	4	5	6	7
Poor		Good		Very Good		Excellent

Scoring

Please add the total score from each question.

For studies with control groups: Scores ≤35 indicate poor quality studies, >35 and ≤45 indicate studies of moderate quality, and >45 indicate good quality studies.

For studies without control groups: Scores ≤32 indicate poor quality studies, >32 and ≤40 indicate studies of moderate quality, and >40 indicate good quality studies.

Supplementary Material Table S2. Baseline demographic and clinical characteristics of rheumatoid arthritis patients receiving tocilizumab treatment from the eligible publications

Parameter	**Wang J et al, 2013*** [9]	**Wang J et al, 2013*** [10] **all trials**	**Enevold C et al, 2014** [20]	**Maldonado-Montoro M et al, 2016** [24]	**Jimenez Morales et al, 2019** [21]	**Maldonado-Montoro M et al, 2018** [23]	**Luxembourger C et al, 2019** [22]
Sample Size (N)	1683	927	79	79	87	77	154
Age (year) [median)(range)] at start with TCZ	52.33±(0.30) (±SD)	52.33±(0.30) (±SD)	54 (22-81)	53.2±12.6	53.4±7.7 (mean±SD)	52.2±11.6	56 ±(13.1) (±SD)
Disease duration (year) [(median) (range)]	9.02±(0.20) (±SE)	9.02±(0.20) (±SE)	10 (0-42)	8 (3-15)	*Not Reported (NR)*	8 (3-15)	14 (7-22)
Male participants [N,%]	21.20%	21.20%	22 (27.8)	14 (17.7)	16 (19.2)	14 (18.2)	28(18.2)
Ethnicity	79.9% white Caucasian	79.9% white Caucasian	Caucasian	Caucasian	Caucasian	Caucasian	91.4% Caucasian & 8.6% North African
Country	14-18 countries including Western Europe, North America & South America	17-18 countries	Denmark	Spain	Spain	Spain	France
ACPA-positive [N, %]	*NR*	*NR*	53 (67.1)	55 (73.3)	59 (69.4)	53 (68.8)	109 (70.8)
Rheumatoid factor [N, %]	76.20%	76.20%	52 (65.8)	52 (65.8)	56 (64.0)	50 (64.9)	116 (75.3)
Joint erosions [N, %]	*NR*	*NR*	57 (72.2)	*NR*	*NR*	*NR*	127 (80.4)
DAS-28-CRP [median)(range)]	*NR*	*NR*	5.3 (3.3-7.3)	*NR*	*NR*	*NR*	5.10 ± 1.28 (mean±SD)
DAS-28-ESR [median)(range)]	6.69±0.02 (mean±SE)	6.69±0.02 (mean±SE)	*NR*	5.6±1.1	5.5±1.2	5.7±1.2	*NR*
Tender joint count (n) [median)(range)]	30.68±0.37 (mean±SE)	30.68±0.37 (mean±SE)	10 (1-26)	9.0 (5.0-14.0)	*NR*	9.0 (5.0-14.0)	*NR*
Swollen joint count (n) [median)(range)]	19.23±0.27 (mean±SE)	19.23±0.27 (mean±SE)	6 (1-23)	4.0 (2.0-7.0)	*NR*	4.0 (2.0-7.0)	*NR*
Serum-CRP (mg/L) [median)(range)]	*NR*	*NR*	15 (0-143)	1.0 (0.4-2.0)	4.9±1.34	1.0 (0.4-2.0)	*NR*
ESR	*NR*	*NR*		30.0 (17.5-49.0)	35.1±28.9	30.3 (17.9-48.5)	*NR*

Parameter	**Wang J et al, 2013*** [9]	**Wang J et al, 2013*** [10] **all trials**	**Enevold C et al, 2014** [20]	**Maldonado-Montoro M et al, 2016** [24]	**Jimenez Morales et al, 2019** [21]	**Maldonado-Montoro M et al, 2018** [23]	**Luxembourger C et al, 2019** [22]
**EULAR-response (good/moderate/none) [N, (%)]**							
Good [N, (%)]	*NR*	*NR*	44 (57.9) at 3 months	*NR*	*NR*	*NR*	84 (54.5) after 3 months
Moderate [N, (%)]	*NR*	*NR*	21(14.5) at 3 months	*NR*	*NR*	*NR*	39 (25.3) after 3 months
None [N, (%)]	*NR*	*NR*	11(27.6) at 3 months	*NR*	*NR*	*NR*	31 (20.1) after 3 months
Satisfactory	*NR*	*NR*	*NR*	54 (69.2) at 6 months; 52 (74.3) at 12 months	*NR*	57 (74.0) at 12 months	*NR*
Unsatisfactory	*NR*	*NR*	*NR*	24 (30.8) at 6 months; 18 (25.7) at 12 months	*NR*	20 (26.0) at 12 months	*NR*
**Concomitant therapy**							
Steroids [N, (%)]	*NR*	*NR*	*NR*	73 (92.4)	93.10%	71 (92.2)	65.60%
MTX [N, (%)]	1,044 (62.0)	1,044 (62.0)	*NR*	35 (44.3)	38 (43.6)	33 (42.9)	47.20%
LEF [N, (%)]	*NR*	*NR*	*NR*	9 (11.4)	*NR*	35 (45.5)	*NR*
**Previous Biologic Therapy (BT)**							
TNFi	*NR*	*NR*	*NR*	30 (38)	60 (69.0)	28 (36.4)	*NR*
TNFi and non-TNFi	*NR*	*NR*	*NR*	24 (30.4)	NA	24 (31.2)	*NR*
Biologic naïve	*NR*	*NR*	*NR*	25 (31.6)	NA	25 (32.5)	*NR*
Number of previous biologic therapy	*NR*	*NR*	*NR*	2.2 (1.0-3.0)	2±1.45	2.2 (1.0-3.0)	*NR*
Duration of the previous biologic therapy (months)	*NR*	*NR*	*NR*	44.5 (16.5-82.0)	41.1±62.2	43.5 (16.5-81.0)	*NR*
**TCZ administration**							
Intravenous (IV)	*NR*	*NR*	*NR*	62 (78.5)	62 (71.2)	60 (77.9)	*NR*
Subcutaneous (SC)	*NR*	*NR*	*NR*	17 (21.5)	25 (28.7)	17 (22.1)	*NR*
Administration dosage and frequency	IV 4 or 8mg/kg every 4 weeks	IV 4 or 8mg/kg every 4 weeks	IV 8mg/kg every 4 weeks (up to 800mg)	IV 8mg/kg or SC 162mg every 4 weeks	IV 8mg/kg or SC 162mg every 4 weeks	IV 8mg/kg or SC 162mg every 4 weeks	*NR*
Remission (DAS28<2.4)	*NR*	*NR*	*NR*	41 (52.6) at 6 months; 41(58.6) at 12 months	*NR*	46 (59.7) at 12 months	*NR*
Low Disease Activity (DAS28<3.6)	*NR*	*NR*	*NR*	55 (70.5) at 6 months; 54 (77.1) at 12 months	*NR*	62 (80.5) at 12 months	*NR*
Shared epitope, % positive	71.0%	71.0%	*NR*	*NR*	*NR*	*NR*	*NR*

NR: Not reported

Supplementary Material Section S2. Quality assessment for the 24 included original studies

Q1: Rationale for study

Please rate the study on the adequacy of the presented hypothesis and rationale.

When rating the study, please consider the following:

- *Was a scientific rationale for chosen genes presented to avoid selective reporting of positive results?*
  If this is a GWAS design, where a hypothesis-free approach is taken, a rationale for selecting this design should be presented.

STUDY	SCORE
Wang J et al., 2013 (GWAS)	7
Wang J et al., 2013	7
Enevold et al., 2014	5
Mar Maldonado-Montoro et al., 2016	7
Jimenez Morales et al., 2018	7
Mar Maldonado-Montoro et al., 2018	7
Luxembourger et al.; 2018	7

Q2: Selection and definition of outcome of interest. The outcome can be cases/disease status or a quantitative trait.

Please rate the study on the classification of the outcome (e.g. disease status or quantitative trait).

When rating the study, please consider the following:

- *Were the cases appropriately defined?*
  Outcome definitions will vary from independent adjudication or reliable laboratorymeasures (strong) to self-report (moderate) to no-description (poor)
- *Were participants appropriately sampled?*
  Participants should be sampled in a way to avoid selection bias as appropriate to the study objectives (e.g. such as selecting the most sick cases if the objective is not to enrich cases). Included participants should reflect the entire population of interest.
- *Were the case/outcome assessors blinded to the genotype status?*

If applicable, was follow-up length appropriate for outcome to occur and was the attrition rate acceptable.

STUDY	SCORE
Wang J et al., 2013 (GWAS)	6
Wang J et al., 2013	7
Enevold et al., 2014	5
Mar Maldonado-Montoro et al., 2016	6
Jimenez Morales et al., 2018	7
Mar Maldonado-Montoro et al., 2018	6
Luxembourger et al.; 2018	7

Q3: Selection and comparability of comparison groups (if applicable)

Please rate the study on appropriateness of comparison groups (e.g. control groups).

When rating the study, please consider the following:

- *Were the controls appropriately defined?*
- *Were the controls sampled in a way to minimize selection bias?*
- *Was a detailed description of selection procedure (i.e. eligibility criteria, sources and methods of ascertainment, methods of matching if applicable) outlined or referenced?*
- *Were the assessors of control status blinded to the genotype status?*

STUDY	SCORE
Wang J et al., 2013 (GWAS)	0
Wang J et al., 2013	0
Enevold et al., 2014	0
Mar Maldonado-Montoro et al., 2016	0
Jimenez Morales et al., 2018	0
Mar Maldonado-Montoro et al., 2018	0
Luxembourger et al.; 2018	0

Q4: Technical classification of the exposure

Please rate the study on the technical classification of the genetic variant.

When rating the study, please consider the following:

- *Was the source (e.g. buffy coat) and method of storage for the DNA sample appropriate?*
- *Were the methods of DNA ascertainment similar for comparison groups (if applicable)?*
- *Was the genotyping platform and allele-calling algorithm appropriate?*
- *Were the genotyping error & call rates appropriate? Call rates below 95% indicate poor genotyping quality.*
- *Were the genotype call rates and SNP missingness similar between the comparison groups?*
- *Was agreement with the Hardy Weinberg equilibrium tested in controls?*

If applicable, did the authors check for samples with outlying heterozygosity to assess quality of genotyping?

STUDY	SCORE
Wang J et al., 2013 (GWAS)	7
Wang J et al., 2013	7
Enevold et al., 2014	3
Mar Maldonado-Montoro et al., 2016	5*
Jimenez Morales et al., 2018	5*
Mar Maldonado-Montoro et al., 2018	5*
Luxembourger et al.; 2018	5

** Saliva samples – extracted using QIAamp DNA Mini Kit (Qiagen GmBH, Hilden, Germany)*

Q5: Non-technical classification of the exposure

Please rate the study on the non-technical classification of the genetic variant.

When rating the study, please consider the following:

- *Did a blinded assessor conduct the genotyping?*
- *Was genotyping conducted in all the participants from the study simultaneously or in smaller batches? If so, were methods across batches same?*

STUDY	SCORE
Wang J et al., 2013 (GWAS)	7
Wang J et al., 2013	7
Enevold et al., 2014	2*
Mar Maldonado-Montoro et al., 2016	6**
Jimenez Morales et al., 2018	6**
Mar Maldonado-Montoro et al., 2018	6**
Luxembourger et al.; 2018	6***

** Genotyping – Luminex platform*
*** Genotyping – real-time PCR using Taq Man probes*
**** Genotyping – allele specific kinetic PCR analysis by LGC-Genomics using the KASPar method*

Q6: Other sources of bias

Please rate the study on the disclosure and discussion of sources of bias.

In addition to selection and classification bias previously discussed, many other potential sources of bias exist (e.g. time-lag bias, attrition bias, et cetera). Please consider whether all sources of bias were disclosed and their effect on the results discussed.

STUDY	SCORE
Wang J et al., 2013 (GWAS)	4
Wang J et al., 2013	5
Enevold et al., 2014	0
Mar Maldonado-Montoro et al., 2016	0
Jimenez Morales et al., 2018	4
Mar Maldonado-Montoro et al., 2018	0
Luxembourger et al.; 2018	6

Q7: Sample size and power

Please rate whether the study was adequately powered.

- *Was the sample size appropriate?*
- *Was an a priori power analysis conducted.*

STUDY	SCORE
Wang J et al., 2013 (GWAS)	3*
Wang J et al., 2013	3*
Enevold et al., 2014	0*
Mar Maldonado-Montoro et al., 2016	1*
Jimenez Morales et al., 2018	3*
Mar Maldonado-Montoro et al., 2018	1*
Luxembourger et al.; 2018	3*

** No sample size calculation reported.*

Q8: A priori planning of analyses

Please rate the study on the planned analyses.

- *Was the analysis plan appropriate and sufficiently described?*
- *Was selective and/or inappropriate reporting avoided (i.e. all results from tests conducted were reported)? Authors should identify where additional results can be found if not included in the primary paper (e.g. supplementary tables).*
- *Were the tested subgroups, interactions, and sensitivity analyses described and reported?*
- *Was the statistical software used identified?*

STUDY	SCORE
Wang J et al., 2013 (GWAS)	7
Wang J et al., 2013	5
Enevold et al., 2014	1
Mar Maldonado-Montoro et al., 2016	3
Jimenez Morales et al., 2018	5
Mar Maldonado-Montoro et al., 2018	3
Luxembourger et al.; 2018	4.5

Q9: Statistical methods and control for confounding

Please rate the study on statistical methods.

- *Were important confounders appropriately controlled?*

- *Were missing data for samples and genetic variant was appropriately handled? >10% missing genotype data is often unacceptable.*
- *Were the results adjusted for multiple testing to avoid false positive results? Please note this is particularly important in analyses of large datasets.*

STUDY	SCORE
Wang J et al., 2013 (GWAS)	3
Wang J et al., 2013	3
Enevold et al., 2014	0
Mar Maldonado-Montoro et al., 2016	0*
Jimenez Morales et al., 2018	1
Mar Maldonado-Montoro et al., 2018	0*
Luxembourger et al.; 2018	5

** Not stated at all*

Q10: Testing of assumptions and inferences for genetic analyses

Please rate the study on the description and test of all assumptions and inferences.

- *Were all assumptions concerning the genetic analysis tested? Specifically,*
  - *i) Haplotypes may be inferred as a result of lack of availability of family data. Numerous methods exist for inferring haplotypes; authors should specifically report how this inference was made.*
  - *ii) In non-family based studies, some individuals may be distantly related or part of a consanguineous group, which may lead to inaccurate results and should be tested with appropriate measures.*

iii) Reported sex and ethnicity should also be checked prior to conducting analyses

STUDY	SCORE
Wang J et al., 2013 (GWAS)	5
Wang J et al., 2013	6
Enevold et al., 2014	5
Mar Maldonado-Montoro et al., 2016	6
Jimenez Morales et al., 2018	5
Mar Maldonado-Montoro et al., 2018	6
Luxembourger et al.; 2018	6

Q11: Appropriateness of inferences drawn from results.

Please rate the study on whether conclusions drawn by the authors were supported by the results and appropriate methods.

STUDY	SCORE
Wang J et al., 2013 (GWAS)	7

Wang J et al., 2013	7
Enevold et al., 2014	3
Mar Maldonado-Montoro et al., 2016	6
Jimenez Morales et al., 2018	6
Mar Maldonado-Montoro et al., 2018	6
Luxembourger et al.; 2018	6

Scoring (for studies without control groups)

STUDY	TOTAL SCORE	QUALITY
Wang J et al., 2013 (GWAS)	56	Good
Wang J et al., 2013	57	Good
Enevold et al., 2014	24	Poor
Mar Maldonado-Montoro et al., 2016	40	Moderate
Jimenez Morales et al., 2018	49	Good
Mar Maldonado-Montoro et al., 2018	40	Moderate
Luxembourger et al.; 2018	55.5	Good

Supplementary Material Table S3. Polymorphisms that were significantly associated with Tocilizumab response in at least one candidate gene study. The results from all available candidate gene studies are presented per variant, including conflicting results.

Study, year	Gene	Polymorphism	Genotype	Responders (n, %)	Non-responders (n, %)	Outcome
Luxembourger et al. 2018	**IL-6R**	**rs12083537**		*EULAR response at 3 months*		*Patients carrying genotype AA was associated with low disease activity response at 12 months using DAS28 score according to ACR criteria*
			AA	70 (87.5)	10 (12.5)	***p= 0.037***
			AG	47 (73.4)	17 (26.6)	
			GG	5 (62.5)	3 (37.5)	
Wang et al. 2013	**IL-6R**	**rs12083537**	NR	NR	NR	*None of the genetic polymorphism in IL-6R showed any association with treatment response*
Maldonado et al. 2018	**IL-6R**	**rs12083537**		*EULAR response at 12 months*		*Patients carrying genotype AA was associated with low disease activity response at 12 months using DAS28 score*
			AA	45 (80.4)	11 (19.6)	–
			AG	12 (60.0)	8 (40.0)	
			GG	0 (0.0)	1 (100.0)	
			A-	57 (75.0)	19 (25.0)	*p= 0.260*
			G-	12 (57.1)	9 (42.9)	*p= 0.076*
Enevold et al. 2014	**IL-6R**	**rs12083537**		*EULAR response at 3 months*		*The major allele AA was associated with poor SJC response at 3 months whereas EULAR response and DAS28-CRP were not significantly affected.*
			AA	(80.0)	(20.0)	
			AG	(93.0)	(7.0)	
Maldonado et al. 2018	**IL-6R**	**rs11265618**		*EULAR response at 12 months*		*Patients carrying genotype CC was associated with low disease activity response at 12 months using DAS28 score*
			CC	38 (80.9)	9 (19.1)	–
			CT	17 (65.4)	9 (34.6)	
			TT	2 (50.0)	2 (50)	
			C-	55 (75.3)	18 (24.7)	*p= 0.276*
			T-	19 (63.3)	11 (36.7)	*p= 0.149*
Maldonado et al. 2018	**IL-6R**	**rs2228145**		*EULAR response at 12 months*		
				23 (76.7)	7 (23.3)	*p= 0.888*
				25 (71.4)	10 (28.6)	
				9 (75.0)	3 (25.0)	
				48 (73.8)	17 (26.2)	*p= 0.876*
				34 (72.3)	13 (27.7)	

Maldonado et al. 2018	**IL-6R**	**rs4329505**		*EULAR response at 12 months*		
			CC	2 (66.7)	1 (33.3)	–
			CT	18 (72.0)	7 (28.0)	
			TT	37 (75.5)	12 (24.5)	
			C-	20 (71.4)	8 (28.6)	*p= 0.902*
			T-	55 (74.3)	19 (25.7)	*p= 1.0*
Enevold et al. 2014	**IL-6R**	**rs4329505**		*EULAR response at 3 months*		*The minor allele CC was associated with poor SJC response at 3 months whereas EULAR response and DAS28-CRP were not significantly affected.*
			CC	(75.0)	(25.0)	
			CT	(78.0)	(23.0)	
			TT	(89.0)	(11.0)	
Morales et al. 2018	***FCGR3A***	**rs396991**		*EULAR response at 12 months*		*Homozygous TT alleles associated with higher EULAR response and greater improvement in DAS28 score*
			GG	10 (83.3)	2 (16.7)	
			GT	30 (66.7)	15 (33.3)	
			TT	24 (88.9)	3 (11.1)	*p= 0.083*
			T	54 (75)	18 (25)	
			G	40 (70.2)	17 (29.8)	
Luxembourger et al. 2018	***FCGR3A***	**rs396991**		*EULAR response at 3 months*		
			GG	16 (72.7)	6 (27.3)	*p= 0.661*
			GT	57 (80.3)	14 (19.7)	
			TT	45 (81.8)	10 (18.2)	
Maldonado et al. 2016	***CD69***	**rs11052877**		*EULAR response at 6 months*		*Carrier of homozygous AA alleles in RA patients achieved remission/low disease activity and improvement of DAS28 score using EULAR response criteria at 6 months*
			AA	21 (87.5)	3 (12.5)	***p= 0.013***
			AG	26 (68.4)	12 (31.6)	
			GG	7 (43.8)	9 (56.3)	
			A-	47 (75.8)	15 (24.2)	***p= 0.030***
			G-	33 (61.1)	21 (38.9)	***p= 0.039***
Luxembourger et al. 2018	**CD69**	**rs11052877**		*EULAR response at 3 months*		
			AA	51 (80.9)	12(19.1)	*p= 0.665*
			AG	56 (81.2)	13(18.8)	
			GG	16 (72.7)	6(27.3)	
Wang et al. 2013	**CD69**	**rs11052877**	NR	NR	NR	

Maldonado et al. 2016	**GALNT18**	**rs4910008**		*EULAR response at 6 months*		*Patients carrying C allele achieved remission/low disease activity and improvement of DAS28 score and higher remission using EULAR response criteria at 6 months (non-significant)*
			CC	14 (87.5)	2 (12.5)	*p= 0.119*
			CT	22 (59.5)	15 (40.5)	
			TT	18 (72.0)	7 (28.0)	
			C-	36 (67.9)	17 (32.1)	*p= 0.919*
			T-	40 (64.5)	22 (35.5)	*p= 0.127*
Luxembourger et al. 2018	**GALNT18**	**rs4910008**	CC	37 (77.1)	11 (22.9)	*p= 0.637*
			CT	61 (83.6)	12 (16.4)	
			TT	25 (78.1)	7 (21.9)	
Maldonado et al. 2016	**CLEC2D**	**rs1560011**		*EULAR response at 6 months*		*Allele G was associated with satisfactory EULAR response at 6 months*
			AA	5 (41.7)	7 (58.3)	*p= 0.058*
			AG	32 (71.1)	13 (28.9)	
			GG	17 (81.0)	4 (19.0)	
			A-	37 (64.9)	20 (35.1)	*p= 0.278*
			G-	49 (74.2)	17 (25.8)	*p= 0.052*
Wang et al. 2013	**CLEC2D**	**rs1560011**	NR	NR	NR	
Maldonado et al. 2016	**ENOX1**	**rs9594987**		*EULAR response at 6 months*		
			AA	17 (81.0)	4 (19.0)	*p= 0.369*
			AG	24 (66.7)	12 (33.3)	
			GG	13 (61.9)	8 (38.1)	
			A-	41 (71.9)	16 (28.1)	*p= 0.566*
			G-	37 (64.9)	20 (35.1)	*p= 0.278*
Wang et al. 2013	**ENOX1**	**rs9594987**	NR	NR	NR	
Luxembourger et al. 2018	**KCNIP1**	**rs703505**		*EULAR response at 3 months*		
			CC	18 (78.3)	5 (21.7)	*p= 0.904*
			CT	56 (78.9)	15 (21.1)	
			TT	49 (81.7)	11 (18.3)	
Wang et al. 2013	**KCNIP1**	**rs703505**	NR	NR	NR	

Morales et al. 2018	**FCGR2A**	**rs1801274**		*EULAR response at 12 months*		
			CC	0 (0)	6 (100)	*p= 1.0*
			CT	4 (15.4)	22 (84.6)	
			TT	4 (40)	6 (60)	
			C	4 (12.5)	28 (87.5)	*p= 0.075*
			T	8 (22.2)	28 (77.8)	*p= 0.576*
Luxembourger et al. 2018	**FCGR2A**	**rs1801274**		*EULAR response at 3 months*		
			CC	35 (81.4)	8 (18.6)	*p= 0.691*
			CT	54 (81.8)	12 (18.2)	
			TT	34 (75.6)	11 (24.4)	
Maldonado et al. 2016	**Non gene**	**rs10108210**		*EULAR response at 6 months*		
			AA	20 (76.9)	6 (23.1)	*p= 0.073*
			AC	19 (55.9)	15 (44.1)	
			CC	15 (83.3)	3 (16.7)	
			A-	39 (65.0)	21 (35.0)	*p= 0.235*
			C-	34 (65.4)	18 (34.6)	*p= 0.435*
Wang et al. 2013		**rs10108210**	NR	NR	NR	
Maldonado et al. 2016	**Non gene**	**rs703297**		*EULAR response at 6 months*		
			CC	12 (63.2)	7 (36.8)	*p=0.182*
			CT	32 (78.0)	9 (22.0)	
			TT	10 (55.6)	8 (44.4)	
			C-	44 (73.3)	16 (26.7)	*p=0.253*
			T-	42 (71.2)	17 (28.8)	*p=0.709*
Wang et al. 2013	**Non gene**	**rs703297**	NR	NR	NR	
Luxembourger et al. 2018	**SLC9A7**	**rs7055107**	NR	NR	NR	
Wang et al. 2013	**SLC9A7**	**rs7055107**	NR	NR	NR	
Wang et al. 2013	**GALNTL4**	**rs4910008**	NR	NR	NR	
Luxembourger et al. 2018	**CD84**	**rs6427528**		*EULAR response at 3 months*		
			AA	–		*p=0.986*
			AG	20 (80.0)	5 (20.0)	
			GG	103 (79.8)	26(20.2)	
	**FCGR3B**	**rs35139848**	CC	76 (82.6)	16 (17.4)	*p=0.548*
			CG	36 (75.0)	12 (25.0)	
			GG	11 (84.6)	2 (15.4)	
	**FCGR2B**	**rs1050501**	CC	3 (100.0)	–	*p=0.739*
			CT	32 (84.2)	6 (15.8)	
			TT	87 (78.4)	24 (21.6)	

	**PTPRC**	**rs10919563**	AA	–	–	*p=0.071*
			AG	26 (92.9)	2 (7.1)	
			GG	97 (77.6)	28 (22.4)	
	**IL10**	**rs1800896**	AA	45 (84.9)	8(15.1)	*p=0.307*
			AG	56 (80.0)	14 (20.0)	
			GG	22 (71.0)	9 (29.0)	
	**TNF**	**rs1800629**	AA	–	–	*p=0.737*
			AG	27 (81.8)	6 (18.2)	
			GG	95 (79.2)	25 (20.8)	
	**IL6**	**rs12700386**	CC	86 (83.5)	17 (16.5)	*p=0.236*
			CG	32 (71.1)	13 (28.9)	
			GG	5 (83.3)	1 (16.7)	
	**IL6**	**rs1800795**	CC	13 (86.7)	2 (13.3)	*p=0.636*
			CG	59 (76.6)	18 (23.4)	
			GG	51 (82.3)	11 (17.7)	
	**IL6**	**rs2069840**	CC	57 (89.1)	7 (10.9)	*p=1.57*
			CG	48 (70.6)	20 (29.4)	
			GG	18 (81.8)	4 (18.2)	
	**TNFRSF10A**	**rs20575**	CC	24 (68.6)	11 (31.4)	*p=0.104*
			CG	57 (80.3)	14 (19.7)	
			GG	42 (87.5)	6 (12.5)	
	**TRAF1/C5**	**rs10818488**	AA	15 (71.4)	6 (28.6)	*p=0.269*
			AG	66 (84.6)	12 (15.4)	
			GG	40 (75.5)	13 (22.5)	
	**TNFRSF1A**	**rs767455**	CC	19 (82.6)	4 (17.4)	*p=0.632*
			CT	60 (82.2)	13 (17.8)	
			TT	44 (75.9)	14 (24.1)	
	**PTPN2**	**rs973767**	AA	94 (79.7)	24 (20.3)	*p=0.411*
			AG	26 (83.9)	5 (16.1)	
			GG	3 (60.0)	2 (40.0)	
	**TGFB1**	**rs1800471**	CC	1 (100.0)	–	*p=0.496*
			CG	18 (90.0)	2 (10.0)	
			GG	104 (78.2)	29 (21.8)	

Supplementary Material Section S3. Meta-analysis results

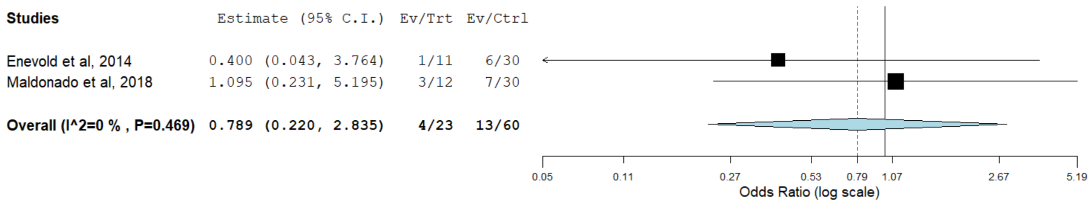

A: IL6R_rs2228145 Homo minor genotype

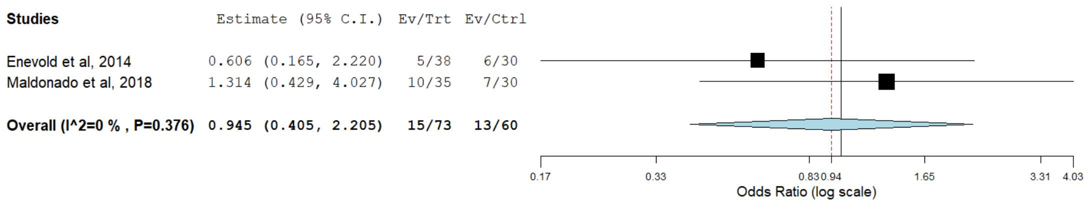

B: IL6R_rs2228145 Hetero genotype

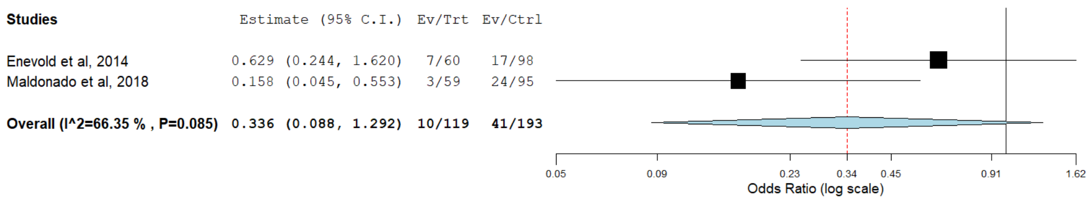

C: IL6R_rs2228145 Allele

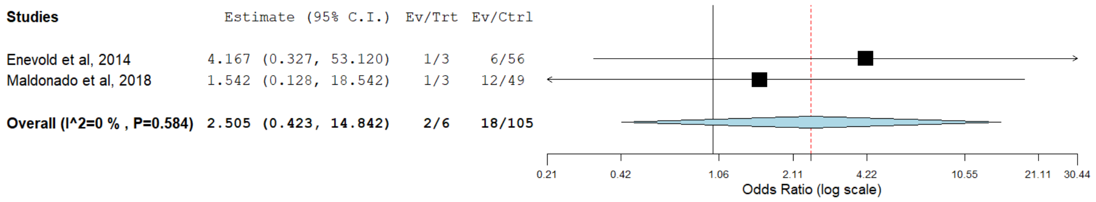

D: IL6R_rs4329505 Homo minor genotype

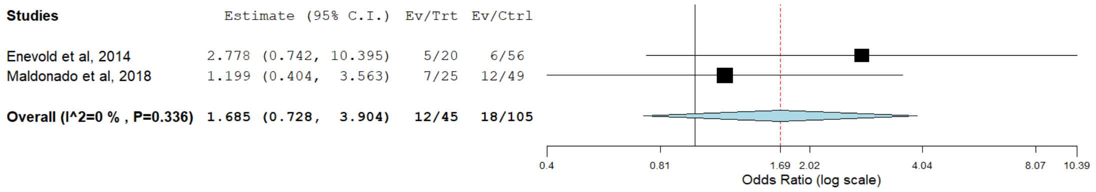

E: IL6R_rs4329505 Hetero genotype

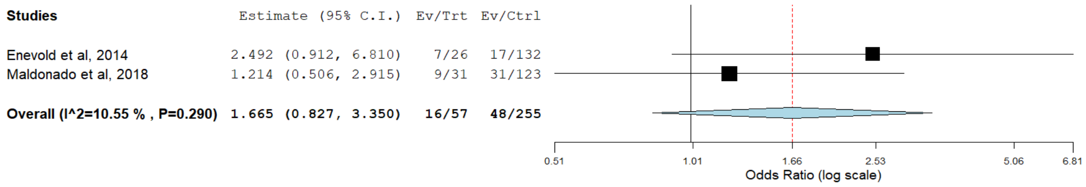

F: IL6R_rs4329505 Allele

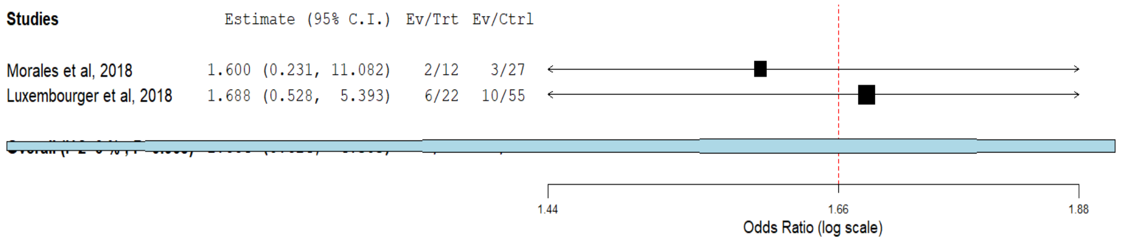

G: FCGR3A_rs396991 Homo minor genotype

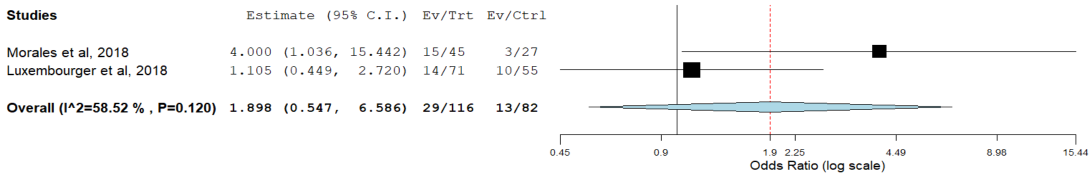

H: FCGR3A_rs396991 Hetero genotype

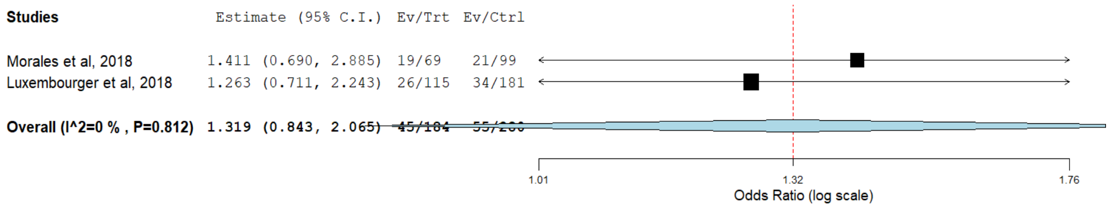

I: FCGR3A_rs396991 Allele

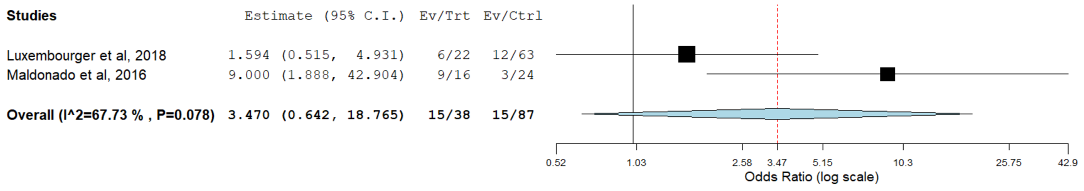

J: CD69_rs11052877 Homo minor genotype

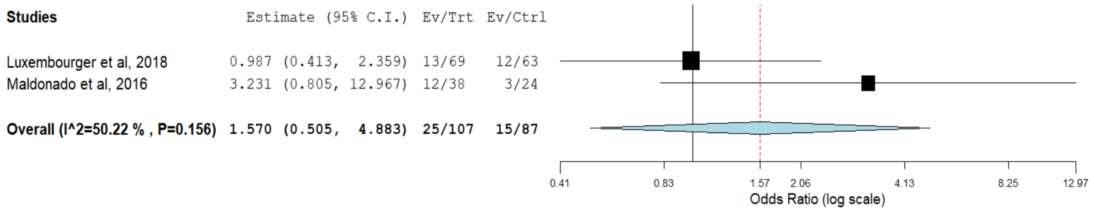

K: CD69_rs11052877 Hetero genotype

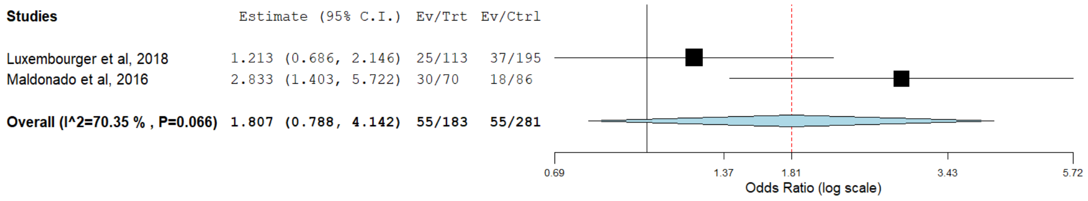

L:CD69_rs11052877 Allele

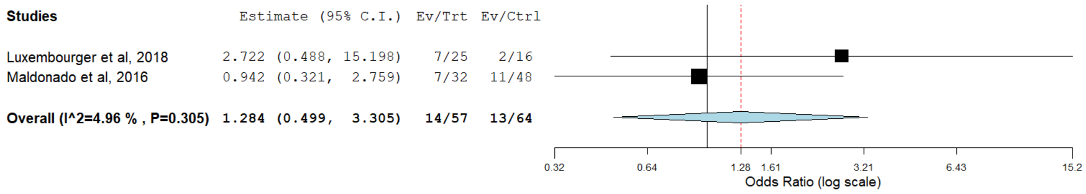

M: GALNT_18_rs4910008 Homo minor genotype

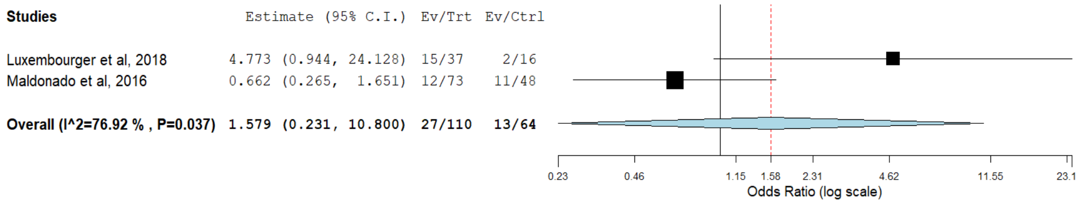

N: GALNT_18_rs4910008 Hetero genotype

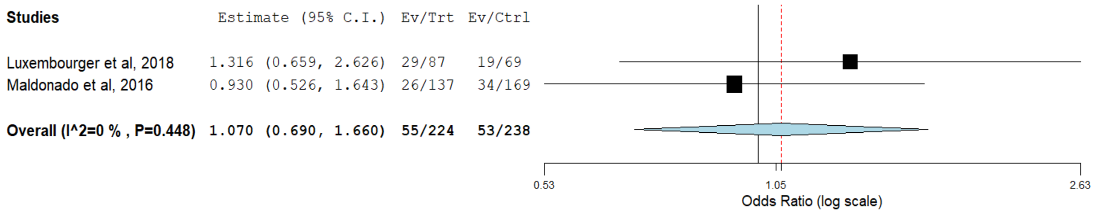

O: GALNT_18_rs4910008 Allele

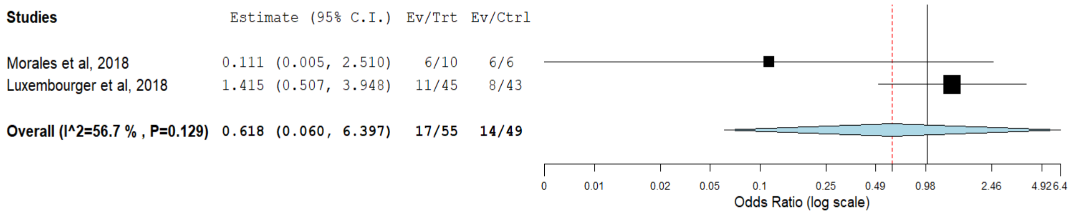

P: FCGR2A_rs1801274 Homo minor genotype

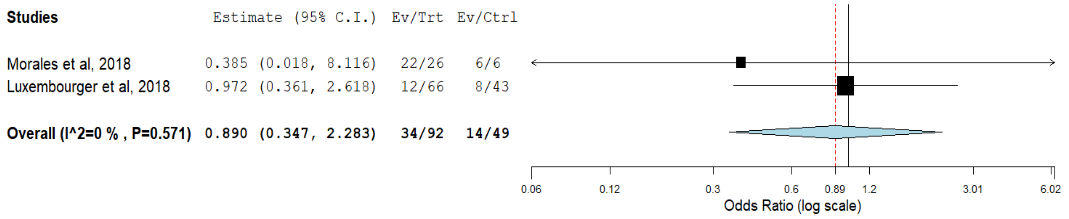

Q: FCGR2A_rs1801274 Hetero genotype

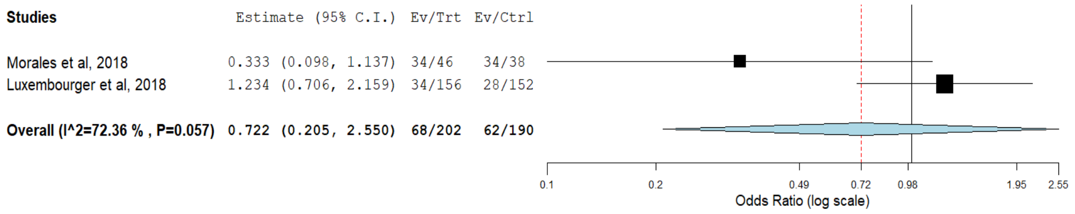

R: FCGR2A_ rs1801274 Allele
